# Incorporating Child Maltreatment Training into Medical School Curricula

**DOI:** 10.1007/s40653-016-0096-x

**Published:** 2016-05-12

**Authors:** Heather L. Pelletier, Michele Knox

**Affiliations:** 1 Department of Psychiatry, University of Toledo, Toledo, OH USA; 2 Department of Psychiatry, Brown University/Hasbro Children’s Hospital, Providence, RI 02903 USA

**Keywords:** Child, Youth, Maltreatment, Abuse, Medical, Student, Training, Efficacy

## Abstract

The Child Advocacy Studies Training (CAST) program has been implemented at the graduate and undergraduate levels, but has not been incorporated in medical school training. This study examines the efficacy of a modified version of the CAST program that is tailored to meet the educational needs of medical students. A total sample of 75 first year medical students (57 at 6 month follow-up) participated in the study and completed analogue vignettes depicting cases of maltreatment. It was hypothesized that, relative to the comparison group, students who completed CAST training would demonstrate better accuracy at identifying and reporting maltreatment. Results indicated that CAST students demonstrated improved accuracy at the conclusion of the CAST program and 6 months later.

Two decades of research has established that the negative outcomes of child maltreatment and other adverse childhood experiences can be vast and chronic (Anda et al. 2006; Flaherty et al. 2013). Short and long term outcomes include poorer mental and physical health and diminished health-related quality of life. Negative mental health outcomes of child maltreatment include anxiety, depression, suicide behavior, aggressive behavior, delinquency, posttraumatic stress disorder, and criminal behavior, among others (Anda et al. 2006; Copeland et al. 2007; Johnson et al. 2002; Rhodes et al. 2013). Negative physical consequences of child maltreatment such as injuries, death, chronic obstructive pulmonary disease, smoking, heart disease, liver disease, and drug use have been identified (Anda et al. 2012; Corso et al. 2008; Repetti et al. 2002; Taylor et al. 2004). Adverse health-related consequences of child maltreatment also include physical disabilities and developmental delays that impede brain development and learning (Scarborough et al. 2009; Sullivan and Knutson 2000).

## Prevention and Intervention

The majority of child maltreatment prevention efforts have involved early education, support, or guidance for those entrusted with caring for young children. Parent training programs have been shown to effectively address parents’ attitudes toward physical discipline, child emotional adjustment, and child-rearing skills and some have evidenced decreased child abuse (Lundahl et al. 2006). However, problems with engagement, retention, and attrition from prevention programs for families create major obstacles to the success of these programs (Connell et al. 2007). Secondary prevention efforts target children or families with one or more risk factors associated with child maltreatment. For example, small to medium effect sizes have been documented for home-visitation programs for families with risk factors for child maltreatment (Eckenrode et al. 2001). One program, known as Safe Environment for Every Kid (SEEK), involves social work support and child abuse prevention training for physicians to address common psychosocial risk factors for maltreatment. This approach has demonstrated moderate effect sizes over 1 year (Dubowitz et al. 2012). Tertiary prevention focuses on families where maltreatment has already occurred, with the goal of reducing the negative consequences of maltreatment and preventing recurrence (US Department of Health and Human Services 1999). These include, for example, mandated reporting laws, health and mental health services for affected children and their families, and parent mentor programs.

## Physicians’ Roles

Physicians have the ability to be effective leaders in many aspects of prevention, intervention, and advocacy addressing child maltreatment (Dubowitz et al. 2011; Reece and Jenny 2005; Reiniger et al. 1995). Even parents who cannot be reached by any other care providers take their children to one or more well-child visits during infancy (Flaherty et al. 2008), and physicians have more frequent contact with maltreated children than other mandated reporters (Flaherty et al. 2008). Accordingly, it is imperative that physicians be adequately equipped to effectively identify and address child maltreatment.

Although mandated reporting holds significant promise for reducing child maltreatment, one of the largest gaps in the tertiary prevention of child maltreatment is low adherence to mandated reporting laws (Flaherty et al. 2002). Research with medical professionals has identified several factors and characteristics that lead to low reporting rates for child maltreatment such as lack of appropriate training, fear of negative consequences to child and family, and concern that there are too few facts to make a report (e.g., Ashton 2004; Feng and Levine 2005; Flaherty et al. 2006; Gunn et al. 2005). Similar factors were identified in a study with medical students (Warner-Rogers et al. 1996). A significant proportion of medical professionals report feeling under-trained and unprepared to identify and report child maltreatment (Flaherty et al. 2000, 2008). The need for more comprehensive training during medical school and residency about the role of psychosocial factors in health has been identified (Astin et al. 2008; Courtois and Gold 2009; Cuff and Vanselow 2004).

Only a few studies have addressed the effectiveness and characteristics of medical training addressing child maltreatment (e.g., Starling and Boos 2003; Reece and Jenny 2005). One study demonstrated that training in effective mandated reporting for mental health professionals resulted in significant improvements in knowledge of reporting laws, accuracy in recognizing child maltreatment, and expertise in reporting (Alvarez et al. 2010). In addition, medical residents who were SEEK-trained became more comfortable and competent in screening for and addressing psychosocial risk factors associated with child maltreatment (Feigelman et al. 2011). One study has examined whether or not training pediatrics residents in child maltreatment using interdisciplinary faculty is effective (Dubowitz and Black 1991). Results of that study indicated significant improvements in residents’ knowledge, skills, and perceived competence in managing cases of child maltreatment. However, similar research has not yet established effective methods for training during medical school.

## Training Content

In order to provide physicians the most effective base of knowledge about child maltreatment, it can be argued that initial training should be integrated into the early medical school curriculum. Training would be especially relevant in medical school since child maltreatment issues are differential diagnoses in the medical conceptualization of many child cases (Flaherty et al. 2008), and this is when medical students begin learning how to conceptualize cases. Early training would provide future physicians time to practice identifying child maltreatment when patients present with complex biomedical and psychosocial factors that may mask signs of maltreatment (Starling and Boos 2003).

It has been suggested that medical training should address risk factors associated with child maltreatment (Reece and Jenny 2005). Examples include established risk and protective factors for the various types of child maltreatment, domestic violence, substance abuse, and family stressors. Medical students need to be informed about signs and symptoms of child maltreatment and state laws about maltreatment. Furthermore, training should address interviewing, screening and assessment skills, aspects of mandated reporting, documentation, and how to effectively work with families and systems of care such as child protective services (Alvarez et al. 2010; Starling and Boos 2003).

### Child Advocacy Studies Training (CAST) Program

The Child Advocacy Studies Training program was developed by the National Child Protection Training Center to educate future professionals to effectively prevent, identify, and respond to child maltreatment. The CAST curriculum is based on the notion that the majority of college graduates will become involved in professions that involve child maltreatment or child advocacy (Vieth et al. 2006). Through CAST, students in a variety of fields work together to become skilled at the prevention, identification, and effective response to child maltreatment. Students are taught how to implement and improve systems of care in the communities they join after graduation. A key component of CAST training is to incorporate both teaching and consultation by professionals in the field (physicians, psychologists, law enforcement, child protective service workers, nurses, prosecutors, etc.). This ensures that the curriculum remains both current and reality-based. The CAST training encourages the use of a highly multidisciplinary approach when addressing the problem of maltreatment, both during and after training. The goal is to steer professionals away from the common problem of several discrete systems that compete for resources and often fail to work together effectively toward community-based solutions (Vieth et al. 2006).

The CAST program has been implemented in a large number of U.S. colleges and universities, but to date has not been widely disseminated to medical schools for the purpose of training future physicians. The current study examines the efficacy of a modified version of the CAST program that is tailored to meet the educational needs of medical students. The CAST medical curriculum was first described in a prior publication (Knox et al. 2013a). Results of prior research on the CAST medical curriculum indicated that first year medical students who were CAST-trained reported feeling significantly more prepared to identify, report, and recommend services for maltreated children compared to medical students who did not receive CAST training (Knox et al. 2013a, b). To further examine the efficacy of the CAST program, research addressing students’ ability to apply what they learn is also needed. This study examines changes in CAST students’ knowledge of child maltreatment and accuracy at identifying and making decisions about reporting child maltreatment as compared to a comparison group of first year medical students who did not complete the training. It was hypothesized that, relative to the comparison group, students who complete CAST training would demonstrate higher total scores (i.e., better accuracy at identifying and choosing to report child maltreatment) on a Vignette Scale (replies to 12 vignettes depicting cases of maltreatment) at post-training, and maintain higher scores 6 months later.

## Method

### Participants

#### Pre-Post Sample Characteristics

Eighty nine students from a total class of 175 first year medical students at a large Midwestern college of medicine stated their intentions to participate and were enrolled in the study. Of these, 40 chose to enroll in the CAST elective course, and all of these students completed both pre-training and post-training measures. The comparison group consisted of 49 students who did not sign up for the elective but agreed to participate in the study. Fourteen students in the comparison group failed to complete the post-training measure for unknown reasons. A total of 75 students completed pre-training and post-training measures and were included in all pre- and post-training analyses. See Table 1 for demographic characteristics of the sample.

**Table 1:** Demographic characteristics by group

	CAST	Comparison
Gender		
Male	23 %	63 %
Female	77 %	37 %
Race		
Caucasian/White	75 %	67 %
Asian	17 %	18 %
Black/African American	0 %	6 %
Hispanic/Latino	0 %	3 %
Other	8 %	6 %
Prior training		
0–1 h	80 %	72 %
2–4 h	15 %	24 %
5–7 h	0 %	2 %
8+ h	5 %	2 %

*N* = 75

#### Characteristics of Sample at Follow Up

A total of 57 students completed measures at pre-training, post-training and 6 months follow-up. Students from the CAST group and the comparison group who did not complete the study measures at all three time points were considered non-completers and were excluded from the final sample analyses.

Of the 57 full study completers, 35 students were in the CAST group and 22 students were in the comparison group; of these 34 were female and 23 were male. The mean age was 23 years (SD = 1.90). There were no significant differences in age or age range between the two groups. Participants in this group ranged in age from 20–29 years old. See Table 2 for demographic characteristics of the final sample.

**Table 2:** Demographic characteristics of final sample by group

	CAST	Comparison
Gender		
Male	26 %	64 %
Female	74 %	36 %
Race		
Caucasian/White	71 %	55 %
Asian	20 %	27 %
Black/African American	0 %	9 %
Hispanic/Latino	0 %	0 %
Other	9 %	9 %

*N* = 57

#### Attrition

The attrition rate for the CAST group was significantly lower than that for the comparison group. For the CAST group, the attrition rate was 0 % from pre to post-training and 12.5 % from post-training to follow-up. For the comparison group, the attrition rate was 28 % from pre to post-training and 55 % from post-training to follow up. Further analyses found no identifiable patterns of attrition. It could be speculated that the comparison group was less invested in the study compared to the students who received a year-long elective course pertaining to the content of the study measures.

### Measures

#### Vignettes

Twelve analog vignettes were written to depict situations likely to occur in medical settings. A variety of forms of child maltreatment, including sexual abuse, physical abuse, neglect, and exposure to domestic violence were depicted in the various vignettes. Furthermore, a variety of victim ages (infancy to adulthood), developmental and physical abilities, and genders were included. After reading each vignette, participants were asked two dichotomous questions about whether the child depicted in the vignette was being maltreated (Yes/No) and whether or not they would report the case to Children’s Services (Yes/No). In order to establish the correctness of the vignette item responses, a panel of three child maltreatment experts was consulted. The panel consisted of a pediatrician who was board certified in Child Abuse Pediatrics, an attorney for the Juvenile Court CASA (Court Appointed Special Advocates) who had been practicing law for 29 years and served as the CASA training coordinator for 13 years, and a social worker for Child Protective Services who had worked on child maltreatment cases for over 20 years. Vignettes were retained if 66.66 % of the experts (two out of three) agreed on responses to *both* of the dichotomous questions asked after each vignette. In cases where there was disagreement, the experts were asked to provide his/her rationale for their decision. Then, the study personnel responsible for writing the vignettes provided the rationale behind the content in the disputed vignette. There were only two vignettes that elicited disagreement (vignettes #3 and #10) and both were discussed. In both cases, the dissenting experts acknowledged that they were unfamiliar with the state’s updated revised code that details laws regarding reporting of child maltreatment for people with disabilities and changed their responses after reviewing the laws. Following this procedure, total inter-rater reliability was 100 % on each of the vignettes; therefore, all vignettes were retained for analyses. Cronbach’s alpha for the vignettes was 0.63. This relatively low alpha coefficient may reflect, in part, the fact that the scale measures a broad diversity of types (e.g., sexual abuse, physical abuse, neglect), and contexts (e.g., victimization reported by a child who is not the victim, victims of different ages and abilities, witnessing violence but not being physically abused oneself) of maltreatment.

#### Accuracy

For each vignette, students’ responses to the question, “Is this child being maltreated?” and, “Would you report this case to Children Services?” were scored as either correct or incorrect. Scores on all dichotomous items were added together, then divided by the total possible score, producing an accuracy score on the vignettes. This was repeated at each of the three time points. The mean accuracy scores for each group, at all three time points, were used in study analyses.

### Procedures

#### CAST Group (Experimental)

All first-year medical students (*N* = 175) enrolled in a college of medicine were contacted by email and invited to participate in a Child Advocacy Elective (CAST) addressing child advocacy and child maltreatment and its consequences, as well as professional responses to child maltreatment. The elective was one of 17 pre-clinical optional elective courses offered to the students on a Credit/No Credit basis. Forty students chose to enroll in the 9-month elective course. The CAST students received an orientation at the beginning of the course and attended didactic presentations to learn and discuss a variety of topics concerning child advocacy. Students also met with faculty for informal, small group mentoring sessions. The course requirements were as follows:

1. Attendance at 2-hour didactics held every other month, during which faculty and related professionals presented relevant topics, viewed documentaries, and facilitated discussion (total of 8 h).
2. Involvement in small group discussions about child maltreatment issues and cases with faculty on alternating months with above (total of 4 to 8 h).
3. Observation of a minimum of one patient on the child and adolescent psychiatric inpatient unit with focus on the impact of adverse life events on children and youths. Students were assigned cases where there was some suspicion or substantiation of current or past child maltreatment. Students interacted with their assigned patients using games and casual discussion only (not clinical interviews). They read the patients’ charts and discussed the cases with inpatient staff members. Students were required to initiate discussion about the cases at the small-group discussion meetings (total of 9 h).
4. Each student studied one de-identified patient case that involved suspected child maltreatment. These were actual patients who were evaluated and treated at the child and hospital’s adolescent psychiatric mental health facility. Students were required to develop and submit a three page paper or give a 20 min presentation about the case they were assigned (total of approximately 8 h).

The same pre-training, post-training, and follow up questionnaires were administered by study personnel (not faculty or other educators) immediately prior to and immediately following students’ completion of the elective and then again 6 months later. Follow up data were collected via email. Students were contacted three separate times to request completion of the follow up measure. For students who did not respond to any of the email reminders, three phone call attempts were made.

#### Comparison Group

Students in the comparison group were first-year medical students who were not enrolled in the CAST Elective. Comparison group participants completed the same battery of self report questions as the CAST group during the same time frame to ensure that all first-year students in the current study were at the same point in their academic curriculum.

## Results

### Descriptive Statistics

Chi square and t-test analyses were used to compare the CAST and comparison groups on the following demographic variables: gender, age, ethnicity, and number of hours of prior training in maltreatment at pre-training and follow-up. At the time of pre-training, the groups differed only on gender χ^2^ (1, *N* = 89) = 14.79, *p* < .001, *Φ* = .41, indicating that there were more males in the comparison group. Similarly, at the time of follow-up, the groups still differed only on gender χ^2^ (1, *N* = 57) = 8.07, *p* < .01, *Φ = .*37, indicating that there were more males in the comparison group. These results represented medium effect sizes. Due to the imbalanced proportion of males to females between groups, all subsequent analyses examined gender as a covariate in order to determine if there was a main effect of gender. Results indicated that there was not a main effect of gender in any of the analyses. In order to determine if groups were equivalent, *t*-test analyses also were used to compare the CAST and comparison groups on all study variables at the time of pre-training. Groups did not differ on any of the study variables.

### Pre-Post Vignette Analysis

Pre-post repeated measures ANOVA analyses examined changes in student knowledge about maltreatment as reflected by total scores on the vignette scale. There was a statistically significant time by group interaction on vignette accuracy, *F*(1,72) = 11.07, *p* < .001, η^2^ = .13. This large effect size favored the CAST group, suggesting a significant increase in vignette accuracy compared to the comparison group from pre to post-training.

### Changes in Student Knowledge Over Time

The next analyses examined changes in student knowledge about maltreatment across all three time points. There was a statistically significant time by group interaction on vignette accuracy, *F*(1,54) = 10.56, *p* < .001, η^2^ = .17. This large effect size favored the CAST group, suggesting a significant increase in vignette accuracy compared to the comparison group over time. Trend analysis illustrates that the CAST group demonstrated greater accuracy on the Vignette Scale at the time of post-training and maintained higher accuracy at the time of follow up compared to the comparison group. See Table 3 below for means and standard deviations of vignette scale scores by group.

**Table 3:** Mean scores for accuracy on the vignette scale by group

Group	Pre-training *M* (SD)	Post-training *M* (SD)	Follow-up *M* (SD)
CAST (*n* = 35)	.77 (.10)	.88 (.07)	.88 (.08)
Comparison (*n* = 22)	.75 (.11)	.73 (.11)	.79 (.10)

*N* = 57

## Discussion

The CAST program for medical students was developed with the general goals of improving child maltreatment knowledge and preparedness and decreasing child maltreatment. Specifically, the CAST program strives to help medical students learn to effectively treat and advocate for the maltreated children they will encounter in practice.

The research hypothesis was confirmed, and analyses demonstrated a moderate short-term effect of the training, with a large effect size at 6-month follow-up. Medical students who enrolled in the CAST program demonstrated improved knowledge about identifying and reporting suspected child maltreatment. These results were observable at the conclusion of the CAST program post-training and suggests that the CAST students’ improved knowledge regarding child maltreatment versus a peer comparison group did not increase due to some other factor (e.g. other medical didactics, life experience) other than the CAST training. Even more importantly, their increased knowledge 6-months after training further highlight the value of the CAST program. These results have important implications for secondary and tertiary prevention efforts related to child maltreatment. Previous research found that medical professionals are among the various groups of professionals who show low adherence to mandating reporting of child maltreatment (Flaherty et al. 2008). The CAST program appears to have potential for addressing these low rates of adherence as students in the CAST program became better at deciding when it was appropriate to report suspected maltreatment.

### Limitations

Results of the current study should be interpreted in light of certain limitations. Although the use of a comparison group was a relative strength of the study, there was no random selection from the medical student population nor was there random assignment to groups. Random assignment would have improved the likelihood of true group equivalency and improved the generalizability of the findings. The groups in the present study were not equivalent with respect to the gender of participants, with the comparison group having a higher proportion of males. Because males have been shown in some studies to be less likely to report maltreatment (Gunn et al. 2005), this may have affected the findings. Further, the lack of random selection increases the likelihood that the students who chose to take the CAST course are not representative of medical students in general. Lastly, there was disproportionate dropout rate in that a larger number of the non-completers were from the comparison group. Future studies should make use of random selection and random assignment to groups.

The fact that the sample was restricted to only one medical school also limits generalizability. Future studies should replicate these findings at multiple sites in a variety of geographical regions and from schools with diverse student populations. Subsequent studies on the CAST program also should evaluate whether the effects last beyond medical school training, and improve actual physician practice related to child maltreatment and child advocacy.

### Implications

Results indicate that the CAST program has the potential to contribute to prevention and intervention efforts within the medical field. This is especially important because research has found that physicians have more frequent contact with maltreated children than other mandated reporters (Flaherty et al. 2008). Although research has repeatedly indicated that medical professionals can be highly effective in the prevention and intervention of child maltreatment (Dubowitz et al. 2011; Reece and Jenny 2005; Reiniger et al. 1995), few attempts to better train future physicians in this area have been studied. The current study has identified the CAST program to be an efficacious approach to begin training future physicians. If the current study’s changes and improvements to students’ knowledge about maltreatment issues could also be achieved with medical students across the country, significant change in child maltreatment in the U.S. could result. The more consistent physicians are in identifying and reporting suspected child maltreatment, the more likely it is that these children and families will receive the necessary intervention needed to protect them from recurring maltreatment. Further, research has shown that maltreated children are at risk for maltreating children in adulthood (Zolotor et al. 2011). Furthermore, children who are victimized have an increased likelihood of future victimization (Cuevas et al. 2010). If fewer children are being maltreated due to effective prevention and intervention efforts, it is likely that cyclical maltreatment and re-victimization will be reduced.

### Future Directions

The results of the current study should be used to further research in this area. It is critical to first determine if the results identified in this study are replicable. Furthermore, while results indicate the current CAST curriculum to be efficacious, some improvements to the program may be beneficial. Although the percentage of correct responses to vignette questions did increase for the CAST group, it is important to note that students in the CAST group did not score 100 % correct. This finding suggests that there is room for improvement in teaching the identification and reporting of child maltreatment. For example, one of the vignettes depicted an 18 year old with a severe developmental disability who experiences neglect. The majority of the total sample responded incorrectly to questions about this vignette at pre-training. Although most CAST students’ responses were more accurate following the CAST program, 12 % still answered incorrectly at the time of post-training. This may suggest a need for more thorough training perhaps with case examples about reporting maltreatment of individuals with disabilities, indicators of neglect, and/or age limits stipulated in state reporting laws.

Further, if a given vignette was endorsed as an example of suspected maltreatment, it should also have been endorsed as worthy of reporting, and therefore rates of identifying and reporting should have been identical. However, there was no one to one correspondence for the CAST-trained group; some cases judged to be maltreatment were not judged to be reportable. This pattern of findings suggests a need to improve teaching about the concept of mandatory reporting of all cases of suspected abuse.

In summary, results of the current study suggest that CAST training in child and adolescent maltreatment and advocacy in the first year of medical school has the potential to improve future physicians’ knowledge and likelihood of effectively identifying and reporting suspected maltreatment. Although replication with additional samples is needed, these findings represent an important first step in establishing improved training in child advocacy at the medical school level. For a copy of the vignettes used in this study, please contact Dr. Heather Pelletier at heather.pelletier@lifespan.org.

## References

[CR1] Alvarez KM, Donohue B, Carpenter A, Romero V, Allen DN, Cross C (2010). Development and preliminary evaluation of a training method to assist professionals in reporting suspected child maltreatment. Child Maltreatment.

[CR2] Anda RF, Felitti VJ, Bremner JD, Walker JD, Whitfield C, Perry BD (2006). The enduring effects of abuse and related adverse experiences of childhood: a convergence of evidence from neurobiology and epidemiology. European Archives for Psychiatry and Clinical Neuroscience.

[CR3] Anda RF, Brown DW, Dube SR, Bremner JD, Felitti VJ, Giles WH (2012). Adverse childhood experiences and chronic pulmonary disease in adults. American Journal of Preventive Medicine.

[CR4] Ashton V (2004). The effect of personal characteristics on reporting child maltreatment. Child Abuse & Neglect.

[CR5] Astin JA, Sierpina VS, Forys K, Clarridge B (2008). Integration of the biopsychosocial model: perspectives of medical students and residents. Academic Medicine.

[CR6] Connell CM, Bergeron N, Katz KH, Saunders L, Tebes JK (2007). Re-referral to child protective services: the influence of child, parent, family and case characteristics on risk factors. Child Abuse & Neglect.

[CR7] Copeland WE, Keeler G, Angold A, Costello J (2007). Traumatic events and posttraumatic stress in childhood. Archives of General Psychiatry.

[CR8] Corso PS, Edwards VJ, Fang X, Mercy JA (2008). Health-related quality of life among adults who experienced maltreatment during childhood. Research and Practice.

[CR9] Courtois CA, Gold SN (2009). The need for inclusion of psychological trauma in the professional curriculum: a call to action. Psychological Trauma: Theory, Research, Practice, and Policy.

[CR10] Cuevas CA, Finkelhor D, Clifford C, Ormrod RK, Turner HA (2010). Psychological distress as a risk factor for re-victimization in children. Child Abuse & Neglect.

[CR11] Cuff PA, Vanselow N (2004). Improving medical education: enhancing the behavioral and social science content of medical school curricula.

[CR12] Dubowitz H, Black M (1991). Teaching pediatric residents about child maltreatment. Journal of Developmental and Behavioral Pediatrics.

[CR13] Dubowitz H, Lane WG, Semiatin JN, Magder LS, Venepally M, Jans M (2011). The safe environment for every kid model: Impact on pediatric primary care professionals. Pediatrics.

[CR14] Dubowitz H, Lane WG, Semiatin JN, Magder LS (2012). The SEEK model of pediatric primary care: can child maltreatment be prevented in a low-risk population?. Academic Pediatrics.

[CR15] Eckenrode J, Ganzel B, Henderson CR, Smith E, Olds DL, Powers J (2001). Preventing child abuse and neglect with a program of nurse home visitation: the limiting effects of domestic violence. Journal of the American Medical Association.

[CR16] Feigelman S, Dubowitz H, Lane W, Grube L, Kim J (2011). Training pediatric residents in a primary care clinic to help address psychosocial problems and prevent child maltreatment. Academic Pediatrics.

[CR17] Feng JY, Levine M (2005). Factors associated with nurses’ intention to report child abuse: a national survey of Taiwanese nurses. Child Abuse & Neglect.

[CR18] Flaherty EG, Sege RD, Binns HJ (2000). Health care providers’ experience reporting child abuse in the primary care setting. Archives of Pediatric and Adolescent Medicine.

[CR19] Flaherty EG, Sege RD, Mattson CL, Binns HJ (2002). Assessment of suspicion of abuse in the primary care setting. Ambulatory Pediatric.

[CR20] Flaherty EG, Sege RD, Price LL, Christoffel KK, Norton DP, O’Connor KG (2006). Pediatrician characteristics associated with child abuse identification and reporting: results from a national survey of pediatricians. Child Maltreatment.

[CR21] Flaherty EG, Sege RD, Griffith J, Price LL, Wasserman R, Slora E (2008). From suspicion of physical child abuse to reporting: primary care clinician decision-making. Pediatrics.

[CR22] Flaherty, E.G., Thompson, R., Dubowitz, H., Harvey, E.M., English, D.J., Proctor, L.J., & Runyan, D.K. (2013). Adverse childhood experiences and child health in early adolescence: ACEs and child health in early adolescence. *JAMA Pediatrics*, 1–8. 10.1001/jamapediatrics.2013.22 PMC3732117 23645114

[CR23] Gunn VL, Hickson GB, Cooper WO (2005). Factors affecting pediatricians’ reporting of suspected child maltreatment. Ambulatory Pediatrics.

[CR24] Johnson RM, Kotch JB, Catellier DJ, Windsor JR, Dufort V, Hunter W (2002). Adverse behavioral and emotional outcomes from child abuse and witnessed violence. Child Maltreatment.

[CR25] Knox, M., Pelletier, H., & Vieth, V. (2013). Effects of medical student training in child advocacy and child abuse prevention and intervention. *Psychological Trauma: Theory, Research, Practice, and Policy*. Advance online publication. doi:10.1037/a0031743

[CR26] Knox M, Pelletier H, Vieth V (2013). Educating medical students about adolescent maltreatment. International Journal of Adolescent Medicine and Health.

[CR27] Lundahl BW, Nimer J, Parsons B (2006). Preventing child abuse: a meta-analysis of parent training programs. Research on Social Work Practice.

[CR28] Reece RM, Jenny C (2005). Medical training in child maltreatment. American Journal of Preventative Medicine.

[CR29] Reiniger A, Robinson E, McHugh M (1995). Mandated training of professionals: a means for improving reporting of suspected child abuse. Child Abuse & Neglect.

[CR30] Repetti RL, Taylor SE, Seeman TE (2002). Risky families: family social environments and the mental and physical health of offspring. Psychological Bulletin.

[CR31] Rhodes AE, Boyle MH, Bethell J, Wekerle C, Tonmyr L, Goodman D (2013). Child maltreatment and repeat presentations to the emergency department for suicide-related behaviors. Child Abuse & Neglect.

[CR32] Scarborough AA, Lloyd EC, Barth RP (2009). Maltreated infants and toddlers: predictors of developmental delay. Journal of Developmental and Behavioral Pediatrics.

[CR33] Starling SP, Boos S (2003). Core content for residency training in child abuse and neglect. Child Maltreatment.

[CR34] Sullivan PM, Knutson JF (2000). Maltreatment and disabilities: a population-based epidemiological study. Child Abuse & Neglect.

[CR35] Taylor SE, Lerner JS, Sage RM, Lehman BJ, Seeman TE (2004). Early environment, emotions, responses to stress, and health. Journal of Personality.

[CR36] US Department of Health and Human Services (DHHS), Substance Abuse and Mental Health Services Administration, Center for Mental Health Services, National Institute of Health, & National Institute of Mental Health (1999). *Mental health: a report of the surgeon general-executive summary*. Rockville, MD. Retrieved August 17, 2011, from http://www.surgeongeneral.gov/library/mentalhealth/toc.html#chapter3

[CR37] Vieth VI, Bottoms BL, Perona AR (2006). Ending child abuse: introducing a collection of new perspectives and practical techniques. Journal of Aggression, Maltreatment & Trauma.

[CR38] Warner-Rogers JE, Hansen DJ, Spieth LE (1996). The influence of case professional variables on identification and reporting of physical abuse: a study with medical students. Child Abuse & Neglect.

[CR39] Zolotor AJ, Theodore AD, Runyan DK, Chang JJ, Laskey AL (2011). Corporal punishment and physical abuse: population-based trends for three-to-11-year-old children in the United States. Child Abuse Review.

